# The role of Life’s Essential 8 and multimorbidity in the risk of new-onset atrial fibrillation: observations from a large prospective cohort study

**DOI:** 10.1097/JS9.0000000000002831

**Published:** 2025-06-24

**Authors:** Junguo Zhang, Shengtao Wei, Ge Chen, Bing Zhang, Yuqin Wang, Zhen-He Huang, Gregory Y.H. Lip, Xiaojie Wang

**Affiliations:** a School of Basic Medical Sciences, Guangzhou University of Chinese Medicine, Guangzhou, China; b Department of Epidemiology, School of Public Health, Sun Yat-sen University, Guangzhou, China; c Department of Geriatrics, Guangzhou First People’s Hospital, Guangzhou Medical University, Guangzhou, China; d Department of Geriatrics, Xiehe Shenzhen Hospital, Huazhong University of Science and Technology, Shenzhen, China; e Liverpool Centre for Cardiovascular Science at University of Liverpool, Liverpool John Moores University and Liverpool Heart & Chest Hospital, Liverpool, United Kingdom; f Department of Clinical Medicine, Aalborg University, Aalborg, Denmark; g Research Team of Prevention and Treatment of Cerebral Hemorrhage Applying Chinese Medicine, The Second Affiliated Hospital of Guangzhou University of Chinese Medicine, Guangzhou, China

**Keywords:** atrial fibrillation, cohort study, life’s essential 8, multimorbidity

## Abstract

**Background:**

The risk of atrial fibrillation (AF) is accentuated by age and comorbidities. Healthy lifestyle behaviors, based on Life’s Essential 8 (LE8), may modify the risk of new-onset AF mitigating the impact of multimorbidity. The aim of this study was to investigate the associations of LE8 with multimorbidity status, linked to AF incidence.

**Methods:**

A total of 255 280 participants free of AF were included. LE8 was assessed using eight metrics and categorized into low, moderate, and high cardiovascular health (CVH). Multimorbidity clusters were determined using latent class analysis using 36 long-term conditions (LTCs), categorized into five groups as follows: three multimorbidity clusters (noncardiovascular disease; mental health; and cardio-metabolic multimorbidity); no LTCs; and singular LTCs. The relationships between LE8 and multimorbidity status with AF incidence were evaluated using Cox models and counterfactual analyses.

**Results:**

During an average follow-up of 11.7 years (SD 2.2), 15 069 participants without AF at baseline (mean age 56.2; SD 8.1; 51.9% female and 48.1% male) developed AF. Individuals with high CVH had a 27% lower risk of AF than those with low CVH. Compared to individuals without multimorbidity, participants with singular LTC or multimorbidity clusters had higher AF risk. Up to 54.7% of the associations between multimorbidity status and AF were mediated by LE8. Counterfactual analysis showed increasing the LE8 score to 80 could reduce AF occurrence in multimorbidity individuals by 22.12% to 24.56%.

**Conclusion:**

Lower LE8 scores and multimorbidity were associated with higher AF incidence. Adherence to a healthy lifestyle, based on LE8 may prevent AF, especially in those with multimorbidity.

## Graphical abstract

**Figure:**
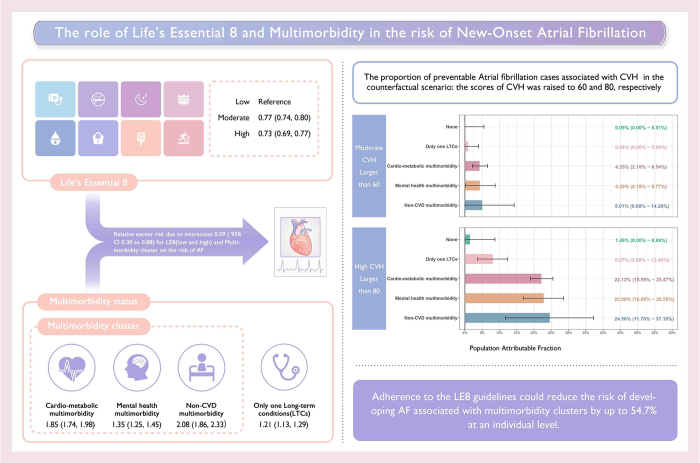

## Introduction

Atrial fibrillation (AF) is the most common heart rhythm disorder, imposing a significant burden on both individuals and health care globally, with an increased mortality and morbidity from stroke, heart failure, dementia, hospitalizations, and health care costs^[1]^.

The risk of developing AF is accentuated by age and comorbidities, leading to clinical complexity in the natural history of AF^[2]^. Multimorbidity, defined as the coexistence of multiple chronic conditions, is an important factor contributing to this clinical complexity^[3]^. AF is more prevalent among individuals with comorbidities, and certain combinations of conditions often cluster together, potentially carrying varying risks for AF^[4,5]^. Consequently, the evaluation and management of multimorbidity are essential in clinical practice, as highlighted by recent guidelines that emphasize addressing cardiovascular risk factors, including lifestyle factors^[6-8]^.

**HIGHLIGHTS**

- Individuals with high cardiovascular health (CVH) based on LE8 had a 27% lower risk of AF.
- Multimorbidity significantly increased AF risk, with up to 54.7% of this risk mediated by LE8 adherence.
- Adhering to a healthy lifestyle (LE8), particularly achieving a score of 80, could substantially reduce the risk of new-onset AF, potentially by up to 24.56%, especially for individuals with multimorbidity.

Unhealthy lifestyle choices and other modifiable risk factors can contribute to an increased risk of AF^[9]^. These factors are often interconnected, and improving one can have a cascading effect, leading to a significant reduction in AF risk by positively impacting other factors^[10]^. To address this, the American Heart Association (AHA) recently updated its cardiovascular health (CVH) metrics, now known as Life’s Essential 8 (LE8), which encompasses four health behaviors and four health factors, thus representing a comprehensive approach to a heart-healthy lifestyle^[11]^.

Healthy lifestyle behaviors, based on LE8, may modify the risk of new-onset AF mitigating the impact of multimorbidity. One recent study reported that a healthier lifestyle is consistently associated with a longer life expectancy across various individual risks and irrespective of the presence of multimorbidity^[12]^. While one study has investigated the association between LE8 and AF incidence^[13]^, there is a gap in the literature regarding the combined effects or interactions between LE8 and multimorbidity (especially the different clusters of comorbidities) in relation to AF risk. Given the intertwined nature of lifestyle factors and chronic diseases, understanding their interactions is crucial for developing effective preventive strategies and reducing the burden of AF.

In light of these gaps, we first aimed to assess the individual and combined effects of LE8 and multimorbidity on AF risk, and second, the mitigating role of LE8 on the associations between multimorbidity and AF. Third, we employed counterfactual analysis to estimate the potential reduction in AF cases attributable to improved LE8. This cohort study has been reported in line with the STROCSS guidelines^[14]^.

## Methods

### Study population

Data obtained from the UK Biobank (Application Number: 69550), a large prospective population-based cohort study, were employed in the current study. The comprehensive study design and participant demographics of the UK Biobank have been previously elucidated^[15]^. In summary, the UK Biobank is a prospective cohort study of over 500 000 individuals in the United Kingdom (aged 40-69 at recruitment), whose baseline data were collected between 2006 and 2010. Demographic information, lifestyle habits, and other potentially health-related data were gathered through a combination of touchscreen questionnaires and face-to-face interviews.

Out of the initial 502 618 participants, exclusions were made for individuals who withdrew from the study (*n* = 1298), those with missing information on sociodemographic and socioeconomic status factors (*n* = 3973), LE8 metrics (*n* = 235 479), and follow-up time data (*n* = 2369). Subsequent to these exclusions, a total of 259 499 participants were deemed eligible for inclusion. For the analysis of AF incidence, individuals with a pre-existing history of AF (*n* = 4219) were additionally excluded, resulting in a final cohort of 255 280 participants (Supplemental Figure 1 http://links.lww.com/JS9/E457).

**Figure 1.**
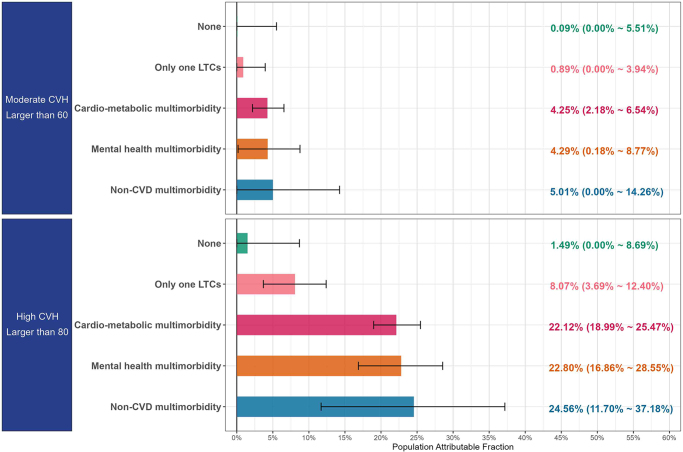
The proportion of preventable atrial fibrillation cases associated with CVH (cardiovascular health) in the counterfactual scenario: the scores of CVH were raised to 60 and 80, respectively. The error bars denote the 95% CI generated by bootstraps.

### Generation of CVH status based on LE8

The AHA’s LE8 included eight metrics: four health behaviors (diet, nicotine exposure, physical activity, and sleep) and four health factors [body mass index, blood pressure [BP], blood glucose, and non-HDL-cholesterol]^[11]^. Each of the eight components was evaluated during the baseline and assigned a score ranging from 0 to 100 points. The LE8 score was calculated by averaging the scores of the eight metrics. Our previous study found a negative association between LE8 and AF incidence, with LE8 scores of 0 and 60 having the highest risk, while the risk was moderate between 60 and 80, and plateauing thereafter^[13]^. In the current study, LE8 scores were classified into three categories: low CVH [0-60), moderate CVH [60-80), or high CVH [80-100].

More detailed definitions and score information for LE8 metrics can be found in Supplementary materials: Supplemental Tables 1 and 2 http://links.lww.com/JS9/E457.

**Table 1:** Baseline characteristics.

Variables	Total	Low CVH	Moderate CVH	High CVH	*P* Value
	(*N* = 255 280)	(*N* = 50 433)	(*N* = 155 370)	(*N* = 49 477)	
Age (years)	56.2 ± 8.1	57.1 ± 7.7	56.8 ± 8.0	53.2 ± 8.2	<0.0001
Sex, n (%)					<0.0001
Male	122 698 (48.1)	28 580 (56.7)	77 516 (49.9)	16 602 (33.6)	
Female	132 582 (51.9)	21 853 (43.3)	77 854 (50.1)	32 875 (66.4)	
Townsend Deprivation Index	−1.4 ± 3.0	−0.7 ± 3.3	−1.5 ± 2.9	−1.8 ± 2.8	<0.0001
Ethnicity, n (%)					<0.0001
White, n (%)	242 710 (95.1)	47 525 (94.2)	147 825 (95.1)	47 360 (95.7)	
Non-White	12 570 (4.9)	2908 (5.8)	7545 (4.9)	2117 (4.3)	
Binge drinking, n (%)					<0.0001
Yes	7458 (2.9)	2850 (5.7)	4189 (2.7)	419 (0.8)	
No	247 822 (97.1)	47 583 (94.3)	151 181 (97.3)	49 058 (99.2)	
Multimorbidity status					<0.0001
None	35 938 (14.1)	2228 (4.4)	19 833 (12.8)	13 877 (28.0)	
Only one LTCs	81 551 (31.9)	12 142 (24.1)	51 982 (33.5)	17 427 (35.2)	
Non-CVD multimorbidity	5233 (2.0)	1335 (2.6)	3097 (2.0)	801 (1.6)	
Mental health multimorbidity	47 077 (18.4)	10 184 (20.2)	27 616 (17.8)	9277 (18.8)	
Cardio-metabolic multimorbidity	85 481 (33.5)	24 544 (48.7)	52 842 (34.0)	8095 (16.4)	
AHA LE8 Scores (out of 100 possible points), Mean ± SD					
Mean Total CVH Score	69.5 ± 11.6	52.4 ± 6.4	70.0 ± 5.5	85.2 ± 4.2	<0.0001
Dietary recommendations for cardiovascular health	61.4 ± 17.3	55.4 ± 16.9	61.6 ± 16.9	67.0 ± 16.9	<0.0001
Physical activity	77.0 ± 37.1	43.7 ± 43.6	81.8 ± 32.9	95.7 ± 14.7	<0.0001
Tobacco/nicotine exposure	76.1 ± 31.9	53.7 ± 39.6	78.5 ± 29.1	91.5 ± 15.6	<0.0001
Sleep health	89.4 ± 18.5	80.4 ± 24.2	90.4 ± 17.2	95.6 ± 11.3	<0.0001
Body mass index	69.8 ± 28.1	46.9 ± 28.7	70.4 ± 25.4	91.2 ± 15.2	<0.0001
Blood lipids (non-HDL cholesterol)	48.1 ± 29.0	34.9 ± 26.7	45.5 ± 26.7	69.5 ± 26.8	<0.0001
Blood glucose	91.3 ± 19.1	79.5 ± 26.0	92.9 ± 17.2	98.5 ± 8.1	<0.0001
Blood pressure	42.9 ± 32.3	25.0 ± 24.4	39.3 ± 29.7	72.7 ± 27.7	<0.0001

CVH, Cardiovascular health; LE8, Life’s Essential 8; SD: Standard Deviation.

**Table 2:** Associations of Life’s Essential 8 (LE8) and multimorbidity status with the incidence of atrial fibrillation.

	Event	IR (95% CI) per 1000 person-years	Crude HR (95% CI)	Adjusted HR (95% CI)	*P*-value
Life’s Essential 8					
Low CVH	4261/50 433 (8.45%)	7.23 (6.49, 7.97)	Ref	Ref	.
Moderate CVH	9116/155 370 (5.87%)	4.88 (4.53, 5.23)	0.67 (0.65, 0.70)	0.77 (0.74, 0.80)	<0.0001
High CVH	1692/49 477 (3.42%)	2.78 (2.32, 3.25)	0.38 (0.36, 0.40)	0.73 (0.69, 0.77)	<0.0001
Per 1-point increase	15 069/255 280 (5.90%)	4.92 (4.64, 5.19)	0.97 (0.97, 0.97)	0.99 (0.98, 0.99)	<0.0001
Multimorbidity status					
None	1125/35 938 (3.13%)	2.55 (2.02, 3.07)	Ref	Ref	.
Only one LTCs	3923/81 551 (4.81%)	3.96 (3.53, 4.39)	1.56 (1.46, 1.67)	1.21 (1.13, 1.29)	<0.0001
Non-CVD multimorbidity	434/5233 (8.29%)	7.18 (4.89, 9.47)	2.86 (2.56, 3.20)	2.08 (1.86, 2.33)	<0.0001
Mental health multimorbidity	1868/47 077 (3.97%)	3.25 (2.74, 3.77)	1.28 (1.19, 1.38)	1.35 (1.25, 1.45)	<0.0001
Cardio-metabolic multimorbidity	7719/85 481 (9.03%)	7.74 (7.15, 8.32)	3.07 (2.89, 3.27)	1.85 (1.74, 1.98)	<0.0001

CI, confidence interval; LTCs: CVH, Cardiovascular health; IR, incident rate; HR, hazard ratio; Long-term conditions.

All results were calculated adjusted by age, sex, ethnicity, Townsend deprivation index, and binge drinking.

Non-CVD multimorbidity mainly included Asthma cancer sclerosis Osteoporosis Thyroid and Tuberculosis.

Mental health multimorbidity cluster mainly included Anxiety and Depression.

Cardio-metabolic mainly included diabetes and Hypertension.

### Defining multimorbidity status and multimorbidity clusters

In this study, multimorbidity is operationally defined as the simultaneous presence of two or more out of a total of 35 long-term conditions (LTCs) in baseline, as derived from established literature sources^[16]^. The selection of these 35 conditions was informed by a comprehensive methodology that incorporated findings from a prominent UK-based investigation^[17]^, a systematic review of existing multimorbidity indices^[18]^, the UK Quality and Outcomes Framework^[19]^, and data accessibility within the UK Biobank. Detailed definitions of the 35 LTCs are presented in Supplemental Table 3 http://links.lww.com/JS9/E457. Anxiety/panic attack, hypertension, and depression were the most common conditions, with prevalences of 54.7%, 53.8%, and 27.5%, respectively (Supplemental Figure 2 http://links.lww.com/JS9/E457).

**Table 3:** Combined effects of Life’s Essential 8 (LE8), multimorbidity status, and the risk of atrial fibrillation.

	Multimorbidity status				
	None	Only one LTCs	Non-CVD multimorbidity	Mental health multimorbidity	Cardio-metabolic multimorbidity
LE8 (HR, 95% CI) a					
High CVH	1.00	1.15 (1.01, 1.31)	2.20 (1.65, 2.93)	1.00 (0.84, 1.19)	1.80 (1.57, 2.05)
Moderate CVH	0.96 (0.85, 1.09)	1.24 (1.12, 1.39)	1.87 (1.58, 2.21)	1.38 (1.22, 1.55)	1.81 (1.62, 2.01)
Low CVH	1.23 (0.98, 1.55)	1.38 (1.22, 1.56)	3.03 (2.52, 3.66)	1.79 (1.57, 2.04)	2.44 (2.19, 2.72)
LE8 (RERI) b					
High CVH					
Moderate CVH		0.10 (-0.05, 0.26)	−0.29 (-0.97, 0.39)	0.39 (0.20, 0.59)	0.04 (−0.16, 0.25)
Low CVH		−0.04 (−0.34, 0.26)	0.75 (−0.12, 1.62)	0.56 (0.23, 0.89)	0.43 (0.11, 0.75)
*P* for multiplicative interaction c < 0.0001					

CI, confidence interval; CVH, Cardiovascular health; HR, hazard ratio; LE8, Life’s Essential 8; RERI, relative excess risk due to interaction.

^a^ All results were calculated adjusted by age, sex, ethnicity, Townsend deprivation index, and binge drinking.

^b^ The estimates of RERI were calculated based on the reference group with high CVH and without multimorbidity.

^c^ Likelihood tests were applied to test the significance of the interaction term by comparing the model with and without the interaction term.

A comprehensive multimorbidity cluster variable was established through latent class analysis utilizing 35 variables related to LTCs, each with binary responses (Yes or No). The analysis, conducted using the poLCA R package, utilized multiple observed categorical variables to derive an unmeasured variable (latent variable) with distinct latent classes. Three latent classes [noncardiovascular disease (non-CVD) multimorbidity, mental health multimorbidity, and cardio-metabolic multimorbidity] were identified, each representing unique multimorbidity clusters based on item-response probabilities. Non-CVD multimorbidity encompasses a range of non-CVDs including asthma, cancer, sclerosis, osteoporosis, thyroid disorders, and tuberculosis. Mental health multimorbidity is primarily characterized by anxiety and depression. Cardio-metabolic multimorbidity centers around metabolic disorders, particularly diabetes and hypertension. The supplemental materials (Supplemental Table 4 and Supplemental Figures 3 and 4 http://links.lww.com/JS9/E457) describe the data collection and latent class analysis in our study.

**Table 4:** Mediation proportion of multimorbidity cluster in the risk of atrial fibrillation attributed to Life’s Essential 8 (LE8).

	Moderate vs. High CVH		Low vs. High CVH		LE8 score	
	Mediation proportion (95% CI)^a^	*P* value	Mediation proportion (95% CI)^a^	*P* value	Mediation proportion (95% CI)^a^	*P* value
Multimorbidity status						
None	-	-	-	-	-	-
Only one LTCs	-c	-b	30.1% (10.7**%,** 60.9%)	0.0019	15.6% (8.7%, 26.2%)	<0.0001
Multimorbidity cluster	2.7% (1.0%, 6.9%)	0.0196	31.9% (25.2%, 39.4%)	<0.0001	23.4% (20.1%, 27.0%)	<0.0001
Non-CVD multimorbidity	-c	-b	11.7% (5.4%, 23.7%)	0.0037	8.5% (4.4%, 15.6%)	0.0009
Mental health multimorbidity	5.2% (1.8%, 13.8%)	0.0258	54.7% (32.6%, 75.0%)	<0.0001	27.3% (19.5%, 36.8%)	<0.0001
Cardio-metabolic multimorbidity	-c	-b	21.4% (15.4%, 29.0%)	<0.0001	19.9% (16.8%, 23.4%)	<0.0001

CI, confidence interval; CVH, Cardiovascular health; LE8, Life’s Essential 8; LTCs: Long-term conditions.

^a^ Adjusted for age, sex, ethnicity, Townsend deprivation index, and binge drinking.

^b^ While the *P* value of the hazard ratio was larger than 0.05, the mediation proportion was not calculated.

^c^ Mediation proportion too small (<1%) to calculate reliably.

Finally, we constructed a five-category variable, referred to as “multimorbidity status,” which includes categories for individuals with no LTC, those with only one LTC, and three distinct multimorbidity clusters.

### Follow-up and AF incident cases

Participants who did not have a prior history of AF at the beginning of the study were monitored from the time of recruitment until the onset of new cases of AF, death, loss to follow-up, or the conclusion of the follow-up period (31 October 2021 for Scotland and 30 September 2021 for England and Wales), whichever came first.

Incidences of new-onset AF were identified through physician diagnoses recorded in primary care databases, hospital admission records, and death registries. AF diagnoses were classified under code I48 according to the International Classification of Diseases, 10th Revision.

### Statistical analyses

Cox proportional hazard models were employed to evaluate the association between multimorbidity status, LE8 [analyzed both continuously (per 1-point increase) and categorically (low, moderate, high)], and incident AF, adjusting for age, sex, ethnicity, binge drinking, and TDI. Details on covariates are in the supplemental methods: covariates.

To explore the combined effect of LE8 and multimorbidity status, a new variable with fifteen categories representing all possible combinations (3 × 5) of LE8 and multimorbidity status was created. HRs and 95% CIs for incident AF were estimated for each category, using the group with low LE8 and no LTCs as the reference. Additive interactions were quantified using relative excess risks due to interaction (RERI) and their 95% CIs^[20]^. Multiplicative interactions were assessed using likelihood ratio tests comparing models with and without the interaction term.

The potential mediating role of LE8 in the relationship between multimorbidity status and AF incidence was examined using the difference method, comparing models with and without LE8^[21]^. Mediation proportions and 95% CIs were calculated. Stratified analyses further investigated the association between LE8 and AF incidence across different multimorbidity status levels.

A counterfactual analysis framework was used to estimate the potential benefit of improving LE8 across different multimorbidity strata^[22]^. Hypothetical LE8 scores of 60 and 80 were set as counterfactual scenarios, with observed scores above these thresholds left unchanged. The difference in AF incidence between observed and counterfactual scenarios represented the preventable AF cases attributable to LE8. Bootstrap resampling (1000 replicates) was used to generate 95% CIs.

To assess potential effect modification by factors known to influence AF risk, subgroup analyses were conducted, stratifying by age, sex, ethnicity, and binge drinking. The robustness of the findings was evaluated through sensitivity analyses: 1) a competing risk analysis considering all-cause mortality as a competing event for AF; 2) exclusion of participants with AF events within the first 2 years of follow-up; 3) additionally adjusted for genetic predisposition and 4) recalculating of LE8 scores by either excluding individual self-reported metrics or omitting all four self-reported metrics collectively. The consistency of the observed associations over time was assessed by examining the effects of LE8 on AF incidence within 5-year time intervals (0-5, 5-10, and ≥10 years) stratified by multimorbidity status. All statistical tests were two-tailed, and a *P*-value < 0.05 was considered statistically significant.

## Results

A total of 255 280 participants were included in this study, with a mean age of 56.2 years (SD) (51.9% women; 95.1% white ethnicity). The participants had a mean LE8 score of 69.5 (SD 11.6) and were divided into high (LE8 scores: [80-100], *n* = 50 433, 19.8%), moderate (LE8 scores: [60-80), *n* = 155 370, 60.8%), and low (LE8 scores: [0-60), *n* = 49 477, 19.4%) CVH groups.

Table 1 displays the baseline characteristics stratified by the LE8 score. Individuals with high CVH tended to be younger, female, and had lower rates of binge drinking. Additionally, they had a higher mean TDI score and appeared less likely to report long-term health conditions. Within the overall statistically significant differences observed in the distribution of multimorbidity statuses across CVH groups (*P* < 0.0001), the proportion of cardio-metabolic multimorbidity was notably lower in the High CVH group (16.4%) compared to the Moderate CVH (34.0%) and Low CVH (48.7%) groups. Within the high CVH group, diet had the lowest mean score (67.0 ± 16.9). In contrast, participants with low CVH had the lowest scores for BP (25.0 ± 24.4) and blood lipids (34.9 ± 26.7).

### Associations of multimorbidity status and LE8 with AF incidence

Over a median follow-up period of 11.66 years (interquartile range 12.45-13.13), AF was observed in 15 069 of the study participants (*n* = 15 069 out of 255 280; incidence rate 5.9%).

Multivariate analysis, adjusting for confounding factors, demonstrated a significant association between CVH and AF incidence (Table 2). Specifically, individuals classified as having high CVH exhibited a 27% lower risk of developing AF compared to their counterparts with low CVH (HR: 0.73; 95% CI: 0.69-0.77). Those with moderate CVH experienced a 23% reduction in AF incidence (HR: 0.77; 95% CI: 0.74, 0.80). Each one-point increment in the LE8 score was associated with a 1% decrease in the risk of AF incidence (HR: 0.99, 95% CI: 0.98, 0.99).

In the current study, 14.1% (*n* = 35 938) reported no LTCs, 31.9% (*n* = 81 551) had one LTC, and 54.0% (*n* = 137 791) experienced multimorbidity at baseline, comprising 2.1% (*n* = 5 233) with non-CVD, 18.4% (*n* = 47 077) with mental health conditions, and 33.5% (*n* = 85 481) with cardio-metabolic multimorbidity. Compared to individuals without multimorbidity, those with singular LTC, non-CVD, mental health issues, and cardiometabolic multimorbidity experienced incrementally higher incidences of AF, with respective HRs and 95% CIs of 1.21 (1.13, 1.29), 2.08 (1.86, 2.33), 1.35 (1.25, 1.45), and 1.85 (1.74, 1.98).

### Interactions between multimorbidity status and LE8 on the incidence of AF

The joint associations between multimorbidity status and LE8 with AF incidence are presented in Table 3 and Supplemental Table 5 http://links.lww.com/JS9/E457.

Compared with participants who had high CVH and without multimorbidity, the risk of AF was significantly higher in those with low CVH and multimorbidity (HR: 2.31, 95% CIs: 2.07, 2.57, Supplemental Table 5 http://links.lww.com/JS9/E457). This increased risk of AF was further observed across specific multimorbidity clusters, including non-CVD, mental health, and cardiometabolic multimorbidity, with HRs ranging from 1.79 to 3.03 (Table 3).

Positive interactions on an additive scale were found between LE8 categories and multimorbidity status regarding AF incidence. For those with low CVH and multimorbidity, the RERI was 0.59 and AP was 0.25, indicating that 25% of AF risk was due to the additive interaction. Specifically, significant additive interactions were also noted for LE8 with specific multimorbidity clusters, including mental health and cardiometabolic multimorbidity (Padditive interactions <0.05). Moreover, multiplicative interaction was found between LE8 and multimorbidity status in relation to the incident AF (Pmultiplicative interactions <0.05).

Subgroup analyses exploring the modifying effect of multimorbidity status on the association between LE8 and AF incidence are presented in Supplemental Table 6 http://links.lww.com/JS9/E457. Compared to individuals without multimorbidity, LE8 exhibited stronger and more significant effects on AF incidence among those with mental health multimorbidity, followed by those with non-CVD multimorbidity and those with cardiometabolic multimorbidity.

Mediation analyses of LE8 on associations of multimorbidity status with AF incidence

When comparing individuals with singular LTC, non-CVD multimorbidity, mental health multimorbidity, or cardiometabolic multimorbidity to those without multimorbidity, the proportions mediated by high CVH relative to low CVH were 30.1% (10.7%, 60.9%), 31.9% (25.2%, 39.4%), 11.7% (5.4%, 23.7%), 54.7% (32.6%, 75.0%), and 21.4% (15.4%, 29.0%) for incident AF, respectively. The estimated mediation proportion of different multimorbidity statuses relative to health, as mediated by the LE8 score, ranged from 8.5% to 27.3% (Table 4, Supplemental Figure 5 http://links.lww.com/JS9/E457).

### Counterfactual analysis

In the counterfactual scenario depicted in Figure 1, the hypothetical elevation of CVH scores to 60 and 80 was modeled to estimate its impact on the incidence of preventable AF. Increasing the CVH score to 80 could significantly reduce the incidence of AF across various participant groups, as follows: 1.49% in those without multimorbidity, 8.07% in individuals with a single LTC, 22.12% in those with cardio-metabolic multimorbidity, 22.80% in individuals with mental health multimorbidity, and 24.56% in those with non-CVD multimorbidity.

### Subgroup and sensitivity analysis

In supplemental Figures 6 and 7 http://links.lww.com/JS9/E457, several subgroup analyses were performed, revealing that the strength of the associations among multimorbidity status, LE8, and AF risk was more pronounced in younger individuals (*P*-interaction < 0.001) and female participants (*P*-interaction < 0.001). Sensitivity analyses revealed no substantial changes in the associations between multimorbidity status, LE8, and AF risk when performing competing risk analyses or excluding AF events occurring within the first 2 years of follow-up, or additionally adjusted for genetic predisposition (Supplemental Table 7 http://links.lww.com/JS9/E457). After omitting all 4 self-reported health behaviors, the HR for High CVH compared to Low CVH was 0.63 (95% CI: 0.51, 0.77). Similarly, omitting individual self-reported metrics resulted in HRs ranging from 0.68 to 0.79 for High CVH, with all associations remaining statistically significant.

The associations between LE8 and incident AF stratified by multimorbidity status exhibited some temporal variations. A significant relationship was observed only within the initial 5 years among individuals with non-CVD multimorbidity. High CVH remained significantly associated with a decreased risk of incident AF for more than 10 years following the initial measurement in groups with a single LTC, mental health conditions, and cardiometabolic multimorbidity. This phenomenon was more pronounced in individuals with multimorbidity (Supplemental Figure 8 http://links.lww.com/JS9/E457).

## Discussion

In this prospective study of over 200 000 community members, we observed that both multimorbidity status and low LE8 score were associated with an elevated risk of AF, and the association of multimorbidity status with AF incidence was mediated by LE8. This suggests that maintaining a healthy lifestyle, which contributes to improved CVH, may be particularly effective in reducing the incidence of AF, especially in individuals with multimorbidity.

This analysis therefore yields four key clinical implications. First, raising the LE8 score to 60 and 80, respectively, could potentially prevent approximately 5% to 25% of AF cases across different multimorbidity clusters. Second, while an unhealthy lifestyle acts synergistically with multimorbidity to significantly increase the risk of developing AF, adherence to the LE8 guidelines could reduce the risk of developing AF associated with multimorbidity clusters by up to 54.7% at an individual level. Third, the temporal association of LE8 with AF risk varies across multimorbidity status, with short-term benefits evident in those with non-CVD multimorbidity and long-term benefits persisting in those with a single LTC, mental health conditions, or cardiometabolic multimorbidity. Of note, the interplay between multimorbidity, LE8, and AF risk is particularly prominent in younger and female individuals, highlighting them as priority populations for intensive CVH promotion and multimorbidity management to prevent AF. Fourth, our findings also bear relevance to surgical settings, where new-onset AF is a frequent postoperative complication, particularly in high-risk patients. The observation that higher LE8 scores are associated with improved surgical outcomes in other contexts – for instance, with reduced surgery-related risks among individuals with inflammatory bowel disease^[23]^ – suggests LE8’s broader potential for preoperative risk stratification. Consequently, LE8 could aid in identifying patients at higher risk for postoperative AF, enabling clinicians to optimize perioperative care and potentially mitigate AF-related surgical complications.

### Comparison with other studies

Multimorbidity is an increasingly important challenge in health care management globally^[24]^, and is linked to an increased risk of AF. Recent work underscores the prevalence of multimorbidity among AF patients, with a majority presenting four or more concurrent LTCs at diagnosis^[5,25]^. Our study shows that even in a population without prevalent AF, multimorbidity affects a substantial 54.0% at baseline.

However, perhaps more crucial is understanding the complex interplay of these conditions, which often manifest as clinically distinct multimorbidity clusters^[26]^. Previous studies focused on multimorbidity clusters in the general AF populations, and assessed the implications of the identified multimorbidity clusters for management and prognosis^[9,26,27]^. One study has investigated the clustering of cardiovascular and renal comorbidities and its association with incident AF, but has not clearly identified specific combinations of LTCs within clusters^[4]^. In the present study, we identified three distinct multimorbidity clusters: non-CVD multimorbidity, mental health multimorbidity, and cardio-metabolic multimorbidity. This reflects the disease complexity associated with AF, and provides valuable insights for risk stratification and prediction, integrating comprehensive clinical data to categorize patients into distinct risk groups^[28]^.

Our findings indicate that cardio-metabolic multimorbidity was most prevalent at baseline within the UK population, followed by mental health multimorbidity. However, non-CVD multimorbidity posed the highest risk for AF, followed by cardio-metabolic multimorbidity. This finding is intriguing, as cardio-metabolic conditions are traditionally considered primary drivers of AF. One potential explanation is that non-CVD multimorbidity, which in our study included conditions such as chronic respiratory diseases and autoimmune disorders, may be associated with higher levels of systemic inflammation and oxidative stress. These pathways are well-established contributors to atrial arrhythmogenesis through structural and electrical remodeling of the atria^[29]^.

While multimorbidity clusters offer a contemporaneous depiction of an individual’s current health status, exploring the influence of LE8, which encompasses modifiable lifestyle factors, provides a prospective viewpoint on mitigating AF risk. The preservation of healthy behaviors is an important strategy that contributes to AF prevention and enhances prognosis in relation to AF management^[30]^. This is important as more than 22% of AF instances could potentially be prevented if individuals maintained optimal CVH^[13]^. In alignment with these findings, the present study has determined that elevated levels of CVH, as assessed by the LE8 metrics, are associated with a significant reduction in AF risk even in different multimorbidity states. Furthermore, the broad nature of multimorbidity means that the observed associations with LE8 and AF risk likely reflect complex underlying interactions, with LE8 potentially exerting varying degrees of influence across different chronic conditions within any given multimorbidity cluster.

Maintaining a healthy lifestyle can substantially lessen the adverse effects of multimorbidity on life expectancy^[12]^. Our mediation analysis underscores the importance of preserving optimal CVH to alleviate the burden of LTCs and multimorbidity, particularly in individuals with mental health multimorbidity. This pronounced effect likely underscores how mental health conditions can negatively influence lifestyle factors integral to LE8, such as diet and physical activity^[31]^. Given that these behaviors are established contributors to AF pathogenesis^[32]^, improving CVH through LE8 adherence could be particularly impactful in mitigating AF risk among individuals with mental health multimorbidity by addressing these modifiable, behaviorally-driven pathways^[33]^.

Moreover, our study uncovers synergistic interactions, both multiplicative and additive, between LE8 and multimorbidity in relation to AF risk. This synergy means that the combined presence of poor LE8 (poor CVH) and multimorbidity results in an AF risk greater than the sum or product of their individual effects. Specifically, a positive additive interaction (RERI = 0.59, AP = 0.25) indicates that 25% of the AF risk in individuals with both poor LE8 and multimorbidity is attributable to this synergistic effect. Also, our counterfactual analysis shows that an increase in the LE8 score to 80 could potentially lead to a significant decrease (>20%) in the occurrence of AF among individuals with multimorbidity, as improving LE8 could mitigate this amplified, synergistic risk. Importantly, LE8 maintained its protective effect against AF incidence beyond 10 years among individuals with mental health or cardio-metabolic multimorbidity. Conversely, an unhealthy lifestyle exacerbates AF risk synergistically with multimorbidity, amplifying physiological stress, worsening underlying conditions, and reducing the effectiveness of treatments^[9,34]^.

These results emphasize the necessity for health care professionals to prioritize lifestyle alterations in the management of multimorbidity and the prevention of AF. Therefore, adherence to LE8 could emerge as a feasible and impactful strategy for public health, effectively reducing AF risk in multimorbidity populations. Furthermore, we found a more pronounced impact of LE8 and multimorbidity status on AF risk in younger and female populations. This could be due to the long duration of exposure to the physiological stress associated with multimorbidity in younger individuals, which may accelerate cardiovascular damage^[35]^, and the potential attenuation of the protective impact of optimal CVH due to age being a primary risk factor for AF^[36]^. Additionally, sex differences could be partially explained by the influence of sex hormones and psychosocial factors^[37]^.

### Strengths and limitations

This study’s major strengths include a large sample size enabling robust statistical analysis, comprehensive evaluation of LE8 and multimorbidity’s complex relationship with AF through multimorbidity status cluster construction, and sensitivity analyses demonstrating the findings’ robustness. Another major strength is the reporting of absolute measures through counterfactual analysis, which quantifies the proportion of AF cases that could potentially be reduced by improving LE8 scores.

However, several limitations warrant acknowledgment. First, given the study’s observational design, the reported associations between LE8, multimorbidity, and AF do not imply causality. The counterfactual analysis, while providing valuable hypothetical estimates of the potential impact of lifestyle changes on AF risk, should be interpreted with caution and not as definitive preventative recommendations. Second, the potential for misclassification inherent in the LCA approach is an unavoidable factor. Third, although diet scores improved with better CVH, the average score in the High CVH group was still below optimal levels. This could be due to limitations in the UK Biobank’s self-reported dietary data or assessment tools, or it may indicate that maintaining a healthy diet remains challenging. This should be kept in mind when interpreting the LE8 diet component findings. Despite some LE8 components relying on self-reported data, which may be biased, our sensitivity analyses (Supplementary Table 8 http://links.lww.com/JS9/E457) indicate that the inverse relationship with incident AF remains even after excluding these metrics, suggesting minimal impact from over-reporting. Additionally, slight changes in HRs when individual metrics are excluded highlight the need to assess all health behaviors and factors collectively, rather than in isolation. Fourth, Temporal changes in LE8 were not accounted for in our analyses. Although prior studies suggest CVH scores are relatively stable or decline^[38]^, future research should examine the influence of LE8 dynamics on AF risk. Fifth, A large number of participants were excluded due to missing data, resulting in a slightly healthier analytical cohort (Supplemental Table 9 http://links.lww.com/JS9/E457). However, these differences were small and unlikely to materially bias the results. Sixth, while we adjusted for several important covariates, including lifestyle factors and socioeconomic status, the selection may not have been exhaustive. There is a possibility of residual confounding from unmeasured or imperfectly measured variables. Lastly, our cohort was predominantly white British, which may limit the generalizability of the findings to other racial or ethnic groups. Differences in lifestyle, genetics, and environmental factors across populations could influence the observed associations, and further studies with more diverse cohorts are needed to explore this.

## Conclusion

Lower LE8 scores and multimorbidity were associated with higher AF incidence. Adherence to a healthy lifestyle, based on LE8 may prevent AF, especially in those with multimorbidity.

## Data Availability

Data are available upon reasonable request to the UK Biobank management team (https://www.ukbiobank.ac.uk/media/px5gbq4q/access_019-access-management-system-user-guide-v4-1.pdf).

## Research registration unique identifying number (UIN)

The project has approval from the North West Multi-centre Research Ethics Committee (MREC) (REC reference: 21/NW/0157).
